# Supplementary material for: Recurrent processes support a cascade of hierarchical decisions
Source: eLife. 2020 Sep 1;9:e56603. doi: 10.7554/eLife.56603 (PMC7513462; doi:10.7554/eLife.56603)
Supplement: Supplementary file 1. — pfc corresponds to the p-value after Bonferroni correction across the five features. Average t-value corresponds to the average t-value in the cluster. [file elife-56603-supp1.docx]

| Feature | Temporal decoding | Spatial decoding | Mass univariate |
| --- | --- | --- | --- |
| Stimulus side | avg t-value = 11.7  41-1500 ms  p < .001  p_fc < .005 | **Left Hemisphere:**  avg t-value = 9.42  p < .001  p_fc < .005  **Right Hemisphere:**  avg t-value = 9.97  p < .001  p_fc < .005 | **Left Hemisphere:**  avg t-value = 2.87  20-1560 ms  p < .001  p_fc < .005  **Right Hemisphere:**  avg t-value = 2.92  40-1560 ms  p < .001  p_fc < .005 |
| Stimulus identity | avg t-value = 7.4  120-845 ms  p < .001  p_fc < .005 | **Left Hemisphere:**  avg t-value = 8.  p < .001  p_fc < .005  **Right Hemisphere:**  avg t-value = 7.8  p < .001  p_fc < .005 | **Left Hemisphere:**  avg t-value = 2.54  100-840 ms  p < .001  p_fc < .005  **Right Hemisphere:**  avg t-value = 2.48  120-860 ms  p < .001  p_fc < .005 |
| Decision | avg t-value = 6.2  150-940 ms  p < .001  p_fc < .005 | **Left Hemisphere:**  avg t-value = 5.54  p < .001  p_fc < .005  **Right Hemisphere:**  avg t-value = 4.1  p < .001  p_fc < .005 | **Left Hemisphere:**  avg t-value = 1.79  210-320 ms  p = .21  p_fc = 1. |
| Uncertainty | avg t-value = 3.  270-1485 ms  p < .01  p_fc < .05 | **Left Hemisphere:**  avg t-value = 2.72  p = .002  p_fc = .01  **Right Hemisphere:**  avg t-value = 3.1  p < .001  p_fc < .005 | **Left Hemisphere:**  avg t-value = 2.42  280-1560 ms  p = .001  p_fc = .005  **Right Hemisphere:**  avg t-value = 2.4  340-1560 ms  p = .008  p_fc = .04 |
| Motor side | avg t-value = 4.9  458-1500 ms  p < .001  p_fc = < .005 | **Left Hemisphere:**  avg t-value = 4.2  p < .001  p_fc < .005  **Right Hemisphere:**  avg t-value = 4  p < .001  p_fc < .005 | **Left Hemisphere:**  avg t-value = 2.56  280-1560 ms  p < .001  p_fc < .005  **Right Hemisphere:**  avg t-value = 2.66  280-1560 ms  p < .001  p_fc < .005 |

**Table 1.** Summary table showing the timing and significance of the results across the five features across the three statistical analyses (temporal decoding, spatial decoding and mass univariate). p_fc corresponds to the p-value after Bonferroni correction across the 5 features. Average t-value corresponds to the average t-value in the cluster.
